# Kinesin superfamily protein 21B acts as an oncogene in non-small cell lung cancer

**DOI:** 10.1186/s12935-020-01323-7

**Published:** 2020-06-12

**Authors:** Zhi-Gang Sun, Feng Pan, Jing-Bo Shao, Qian-Qian Yan, Lu Lu, Nan Zhang

**Affiliations:** 1 Department of Thoracic Surgery, Central Hospital Affiliated to Shandong First Medical University, Jinan, 250013 People’s Republic of China; 2 Department of Ethics Committee, Central Hospital Affiliated to Shandong University, Jinan, 250012 People’s Republic of China; 3 Weifang Medical University, Weifang, 261053 People’s Republic of China; 4 Department of Oncology, Jinan Central Hospital, Cheeloo College of Medicine, Shandong University, Jinan, 250012 People’s Republic of China; 5 Shandong First Medical University, Jinan, 250013 Shandong China; 6 Department of Oncology, Central Hospital Affiliated to Shandong First Medical University, Jinan, 250013 People’s Republic of China

**Keywords:** Non-small cell lung cancer, KIF21B, Proliferation, Oncogene

## Abstract

**Background:**

Kinesin superfamily proteins (KIFs) serve as microtubule-dependent molecular motors, and are involved in the progression of many malignant tumors. In this study, we aimed to investigate the expression pattern and precise role of kinesin family member 21B (KIF21B) in non-small cell lung cancer (NSCLC).

**Methods:**

KIF21B expression in 72 cases of NSCLC tissues was measured by immunohistochemical staining (IHC). We used shRNA-KIF21B interference to silence KIF21B in NSCLC H1299 and A549 cells and normal lung epithelial bronchus BEAS-2B cells. The biological roles of KIF21B in the growth and metastasis abilities of NSCLC cells were measured by Cell Counting Kit-8 (CCK8), colony formation and Hoechst 33342/PI, wound-healing, and Transwell assays, respectively. Expression of apoptosis-related proteins was determined using western blot. The effect of KIF21B on tumor growth in vivo was examined using nude mice model.

**Results:**

KIF21B was up-regulated in NSCLC tissues, and correlated with pathological lymph node and pTNM stage, its high expression was predicted a poor prognosis of patients with NSCLC. Silencing of KIF21B mediated by lentivirus-delivered shRNA significantly inhibited the proliferation ability of H1299 and A549 cells. KIF21B knockdown increased apoptosis in H1299 and A549 cells, down-regulated the expression of Bcl-2 and up-regulated the expression of Bax and active Caspase 3. Moreover, KIF21B knockdown decreased the level of phosphorylated form of Akt (p-Akt) and Cyclin D1 expression in H1299 and A549 cells. In addition, silencing of KIF21B impeded the migration and invasion of H1299 and A549 cells. Further, silencing of KIF 21B dramatically inhibited xenograft growth in BALB/c nude mice. However, silencing of KIF21B did not affect the proliferation, migration and invasion of BEAS-2B cells.

**Conclusions:**

These results reveal that KIF21B is up-regulated in NSCLC and acts as an oncogene in the growth and metastasis of NSCLC, which may function as a potential therapeutic target and a prognostic biomarker for NSCLC.

## Background

As an important part of the cytoskeleton, the dynamic behavior of microtubules plays an important function in many biological processes, including cell motility, intracellular trafficking and mitotic spindle formation. Recently, emerging studies has reported that abnormal changes in microtubule dynamics are frequently observed during cancer cell divisions, leading to chromosomal instability, aneuploidy or drug resistances [1]. Currently, some effective anti-cancer drugs are microtubule interfering drugs such as the taxanes [2]. However, despite all these studies, the precise role of microtubules in the development of cancer is still poorly studied.

Kinesin superfamily proteins (KIFs) serve as microtubule-dependent molecular motors, play a fundamental role in cellular functions though transport certain cellular proteins, macro-molecules, and organelles [3, 4]. Recently, it has been reported that KIFs functions in mitotic cell division by participating in chromosomal and spindle movement, indicating that deregulation of KIFs may be associated with tumorigenesis [5–7]. Kinesin family member 21B (KIF21B), located at 1q32.1, is the motor of Kinesin-4 motor, and contains a motor domain, a stalk and a tail domain which binds to microtubules. It has been confirmed that KIF21B, a classic kinesin protein, could function as a regulator of microtubule dynamics [8]. It could inhibit the growth of microtubules through its tail domain, acting as a potential microtubule-pausing factor [9, 10]. As well known, microtubules and kinesin proteins play critical roles in intercellular signal transduction, malignancy, tumor progression and metastasis [11]. Arai et al. showed that high KIF21B expression was correlated with poor disease-free survival rate of prostate cancer patients [12]. Zhao et al. revealed that KIF21B expression was up-regulated and associated with prognosis in hepatocellular carcinoma [13]. However, the precise role of KIF21B in carcinogenesis of non-small cell lung cancer (NSCLC) remains unclear.

In this study, we aimed to detect the expression of KIF21B in NSCLC, and investigate its functional role in the progression of NSCLC. Our data demonstrated that KIF21B was up-regulated in NSCLC, and high KIF21B expression was associated with advanced clinicopathologic features of NSCLC. Importantly, KIF21B was an independent factor for the 5-year survival rate of NSCLC patients. The loss-of-function experiments suggested that KIF21B exerted an oncogenic role in the growth and invasion of NSCLC cells.

## Material and method

### Patients and tissue specimens

A total of NSCLC samples (n = 72) and adjacent normal tissues (n = 20) in the study were collected from NSCLC patients enrolled in Jinan Central Hospital from January 2011 to December 2014. The inclusion criteria of the samples in this study were as follows: (1) patients undergo radical surgery with no seriously surgical contraindications, (2) stage I–IIIa NSCLC patients, and (3) No radiotherapy or chemotherapy before surgery.

### Follow-up

After operation, 62 patients had chemotherapy, 27 patients had radiotherapy, and 32 patients received therapy of EGFR-TKI. We recorded the location and tumor recurrence time, and survival was measured.

### Cell culture and infection

The human NSCLC cell lines H1299 and A549 and normal lung epithelial bronchus BEAS-2B cells were obtained from Chinese Academy of Sciences Cell Bank (Shanghai, China). H1299 and A549 cells were cultured in DMEM (Hyclone, USA) supplementing with 10% FBS (Gibco, Thermo Fisher, USA), penicillin (100 U/mL; Sigma Aldrich, Germany) and streptomycin (0.1 mg/ml; Sigma Aldrich) at 37 °C. BEAS-2B cells were cultured in RPMI 1640 (Gibco, USA) supplementing with 5% FBS. The recombinant lentiviruses packaged with shRNA-KIF21B were obtained from Genechem (Shanghai Genechem Co., Ltd.). The target sequence of KIF21B was as follows:5′-GGA GCT GAT GGA GTA TAA G-3′;The sequence of shRNA-NC control was as follows:5′-TTC TCC GAA CGT GTC ACG T-3′.

### Immunohistochemistry (IHC)

The paraffin-embedded samples were sectioned at 4 μm and the expression of KIF21B was evaluated by immunohistochemistry using the EliVisionTMplus kit (Maixin, Fuzhou, China) with anti-KIF21B antibody (ab121931; Abcam, UK) according to the instructions provided. Two observers who were blinded with respect to the information of patients and tumor grade were performed quantification. In each case, 10 fields were randomly selected under the light microscope, and the expression level of KIF21B was evaluated by staining intensity and proportion of positive stained cells. The staining intensity was scored strong staining (3), moderate staining (2), weak staining (1) and no staining (0). The proportion of positive stained cells was graded 0–10% (0), 11–25% (1), 26–50% (2), 51–75% (3), and > 75% (4). The multiplication of the scores of the two groups was the final score of the expression level of KIF21B, and the score less than or equal to 6 was the low expression group, and the score more than 6 was the high expression group [14, 15].

### Reverse transcription polymerase chain reaction (RT-PCR) analysis

We extracted total RNA from cells by an RNA extraction kit (CWBIO, Beijing, China). The reverse transcription was then conducted using a HiFiScript cDNA Synthesis Kit (CWBIO). KIF21B mRNA expression was measured by a SYBR Premix Ex Taq II kit (Takara, Japan). The obtained data was analyzed using 2^−ΔΔCt^ method. β-actin was used as the control. The primers used in this study were synthesized by GENEWIZ (Suzhou, China) and as follows: KIF21B forward, 5′-CGA GGA GAC GGA TGA GAA CG-3′; reverse, 5′-CCA CCA GGC TCT CTT CAC TG-3′; β-actin forward, 5′-CCC GAG CCG TGT TTC CT-3′, reverse, 5′-GTC CCA GTT GGT GAC GAT GC-3′.

### Western blot

After being infected for 72 h, cells were lysed in RIPA Buffer (CWBIO) and quantified using a BCA kit (CWBIO). Proteins were separated with 10% SDS-PAGE gel and then electrotransferred to PVDF membranes (Millipore, MA, USA). Subsequently, we blocked the membranes in 5% nonfat milk for 1 h, and then incubated them with primary antibodies at 4 °C overnight. After that, the membranes were incubated with secondary antibodies (dilution, 1:3000; Proteintech Group) for 1 h at room temperature. The protein signals were developed using an ECL kit (CWBIO) and analyzed using Image J software (NIH, USA) [16].

### Cell counting kit-8 (CCK8) assay

1 × 10^3^ cells transfected with shRNAs were seeded in a 96-well plate at 37 °C for 1, 24, 48 and 72 h, respectively. Cells were then incubated with 10 μl of CCK8 solution (Solarbio Science & Technology, Beijing, China) for another 1 h at 37 °C. Finally, a plate reader determined the OD value at 450 nm.

### Colony formation assay

Cells infected with shRNA-KIF21B lentivirus (200 cells/well) were plated in a 6-well plate and cultured for 1–2 weeks. After being fixed with 4% paraformaldehyde for 30 min, the cells were stained with 0.1% crystal violet for another 30 min. Finally, we counted the number of colonies.

### Hoechst 33342/PI assay

Cells infected with shRNAs (1 × 10^3^ cells/well) were cultured in a well plate for 24 h. Then, we co-stained the cells with Hoechst 33342 (10 μg/mL; Beyotime, Shanghai, China) and PI (5 μg/mL; Beyotime) for 15 min. After washing with PBS for 3 times, cell fluorescence was observed and captured under a fluorescence microscope.

### Wound-healing assay

Following infection of 24 h, the cells (5 × 10^5^ cells/well) were cultured in a 6-well plate for 12 h. A wound was generated using a pipette tip in each well. After 24 h of culture, the wound closure was measured.

### Transwell assay

Transwell chambers (Millipore) coated with Matrigel was carried out to assess cell invasion, whereas cell migration assay did not add Matrigel. We plated about 1 × 10^5^ lentivirus-infected cells in the upper chamber; the lower chamber was filled with medium containing FBS. Subsequent to a period of 24 h, we used cotton swabs to wipe out the cells that were not through the membrane. We fixed the migrated or invaded cells in 4% paraformaldehyde for 30 min, and then stained them with 0.1% crystal violet for another 20 min. The number of migrated or invaded cells was quantified under a microscope (magnification, × 100) in five random microscope fields.

### In vivo tumorigenic assay

According to the NIH guidelines, animal procedures were carried out. Ten BALB/c female nude mice (20 ± 2 g) were upraised in barrier facilities on a 12 h light/dark cycle and randomly divided into 2 groups (n = 5). H1299 cells were infected with lentivirus-shRNA-KIF21B or lentivirus-shRNA-control for 48 h. The H1299 cells (5 × 10^6^) were subcutaneously implanted in the right axils of nude mice. All nude mice were euthanized 5 weeks following injection, the tumors were removed and their weights were measured.

### Statistical analysis

The measurement data were presented as Mean ± SD and analyzed using Student’s *t* test or one-way ANOVA. The enumeration data were analyzed using Fisher’s exact probability test. The Kaplan–Meier with log-rank test was performed for survival rate. Cox proportional hazard model was performed for multivariate analysis. We used SPSS (IBM SPSS Statistics 25, USA) to perform for statistical analysis. And P < 0.05 was regarded as significant difference.

## Results

### KIF21B is up-regulated in NSCLC tissues

**Fig. 1:**
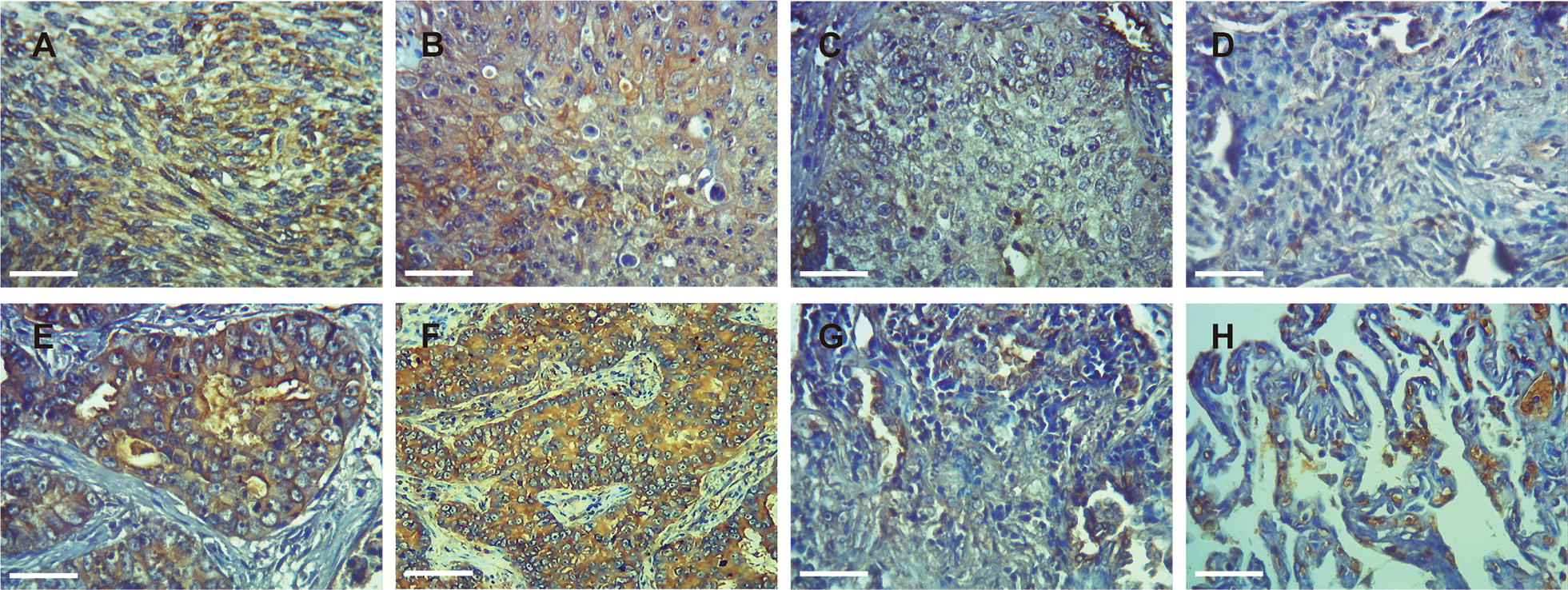
Immunohistochemical staining of lung cancer tissue sections demonstrating KIF21B (original magnification × 400). **a, b** Lung squamous cell carcinoma specimen with high expression of KLF6-SV1. **c** Lung squamous cell carcinoma specimen with low expression of KLF6-SV1. **d** The corresponding normal lung tissue specimen with no KLF6-SV1 expression (Control). **e**, **f** Lung adenocarcinoma specimen with high expression of KLF6-SV1. **g** Lung adenocarcinoma specimen with low expression of KLF6-SV1. **h** The corresponding normal lung tissue specimen with no KLF6-SV1 expression (Control). The scale bar was 50 μm

**Fig. 2:**
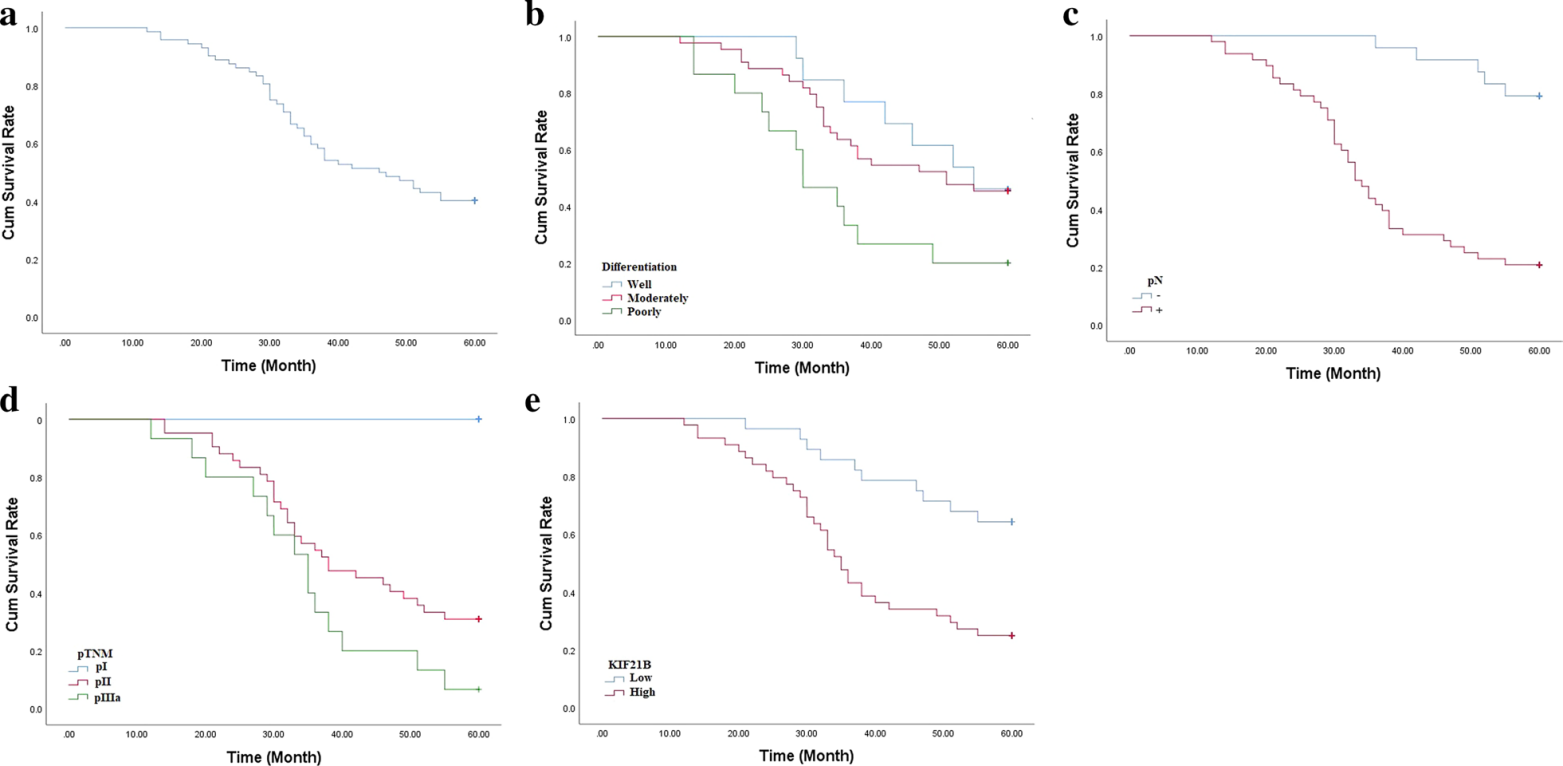
**a** The Kaplan–Meier survival curve of 72 cases of NSCLC patients. **b** Survival curves of NSCLC patients with tumor differentiation. **c** Survival curves of NSCLC patients with negative or positive pN. **d** Survival curves of NSCLC patients with different pTNM. **e** Survival curves of NSCLC patients with low or high expression of KIF21B expression

As indicated by IHC assay (Fig. 1), we found that the positive KIF21B signal was mainly located in cytoplasm. KIF21B expression in the NSCLC tissues (44/72, 61.1%) was significantly higher than that in adjacent normal lung tissues (0/20, 0.0%) (*P *< 0.01, Fig. 1a–h). The high expression of KIF21B was correlated with pN (*P *< 0.05) and pTNM stage (*P *< 0.05) (Table 1). Moreover, the 5-year survival rate of the 72 cases of NSCLC was 40.3%, which was significantly correlated with tumor differentiation (*P *< 0.05), pN (*P *< 0.01), and pTNM stage (*P *< 0.01) (Fig. 2a–d and Table 2). Importantly, NSCLC patients with higher KIF21B expression had poor prognosis (*P *< 0.01) (Fig. 2e and Table 2). Further, pN and KIF21B were independent factors for the NSCLC patients’ 5-year survival rate (Table 3).

**Table 1:** Correlation between KIF21B expression and clinicopathological features of the patients with non-small cell lung cancer

Clinical features	Patients	KIF21B expression		
		Low	High	*P* Value^a^
	72	28	44	
Gender				0.449
Male	40	14	26	
Female	32	14	18	
Age, year				0.978
< 60	31	12	19	
≥ 60	41	16	25	
Smoking				0.611
No	48	20	28	
Yes	24	8	16	
Histological type				0.214
SCC	32	15	17	
ADC	40	13	27	
Differentiation				0.145
Well	13	8	5	
Moderately	44	16	28	
Poorly	15	4	11	
pT				0.404
pT1	11	5	6	
pT2	51	21	30	
pT3	10	2	8	
pN				0.017
−	24	14	10	
+	48	14	34	
pTNM				0.031
pI	15	9	6	
pII	42	17	25	
pIIIa	15	2	13	

*SCC* squamous cell carcinoma, *ADC* adenocarcinoma, *pT* tumor size, *pN* lymph node metastasis, *pTNM* tumor stage

*P* Value^a^: Fisher’s exact probability test

**Table 2:** Univariate analysis with respect to 5-year survival of the patients with non-small cell lung cancer

Clinical features	Patients	5-year survival (%)		*P* Value^b^
		Patients	Rate (%)	
	72	29	40.3	
Gender				0.352
Male	40	15	37.5	
Female	32	14	43.8	
Age, year				0.624
< 60	31	12	38.7	
≥ 60	41	17	41.5	
Smoking				0.182
No	48	22	45.8	
Yes	24	7	29.2	
Histological type				0.115
SCC	32	9	28.1	
ADC	40	20	50.0	
Differentiation				0.035
Well	13	6	46.2	
Moderately	44	20	45.5	
Poorly	15	3	20.0	
pT				0.069
pT1	11	8	72.7	
pT2	51	20	39.2	
pT3	10	1	10.0	
pN				0.001
−	24	19	79.2	
+	48	10	20.8	
pTNM				0.001
pI	15	15	100.0	
pII	42	13	31.0	
pIIIa	15	1	6.7	
Chemotherapy				0.893
No	10	4	40.0	
Yes	62	25	40.3	
Radiotherapy				0.801
No	45	18	40.0	
Yes	27	11	40.7	
EGFR-TKI therapy				0.788
No	40	17	42.5	
Yes	32	12	37.5	
KIF21B				0.001
Low	28	18	64.3	
High	44	11	25.0	

*SCC* squamous cell carcinoma, *ADC* adenocarcinoma, *pT* tumor size, *pN* lymph node metastasis, *pTNM* tumor stage, *EGFR-TKI* growth factor receptor-tyrosine kinase inhibitor

*P* Value^b^: Log-rank test

**Table 3:** Results of cox regression multivariate 5-year survival analysis of the patients with non-small cell lung cancer

	B	SE	Wald	P	HR	95.0% CI for HR
Gender	0.573	0.409	1.968	0.161	1.774	0.796–3.951
Age	− 0.332	0.372	0.796	0.372	0.718	0.346–1.488
Somking	− 0.079	0.467	0.029	0.865	0.924	0.370–2.307
Histological type	− 0.755	0.436	1.335	0.248	1.424	0.782–2.594
Differentiation	0.353	0.306	2.757	0.097	1.707	0.908–3.209
pT	0.455	0.390	1.357	0.244	1.576	0.733–3.387
pN	1.948	0.656	8.805	0.003	7.011	1.937–25.379
pTNM	0.055	0.401	0.019	0.890	1.057	0.482–2.318
Chemotherapy	− 0.749	0.576	1.692	0.193	0.473	0.153–1.462
Radiotherapy	0.257	0.381	0.455	0.500	1.293	0.613–2.726
EGFR-TKI therapy	− 0.072	0.503	0.020	0.8587	0.931	0.348–2.493
KIF21B	0.900	0.439	5.081	0.0040	2.460	1.041–5.811

*B* regression coefficient, *SE* standard error, *Wald* Wald value, *HR* hazard ratio, *CI* confidence interval

### Silencing of KIF21B impedes the proliferation of NSCLC cells

**Fig. 3:**
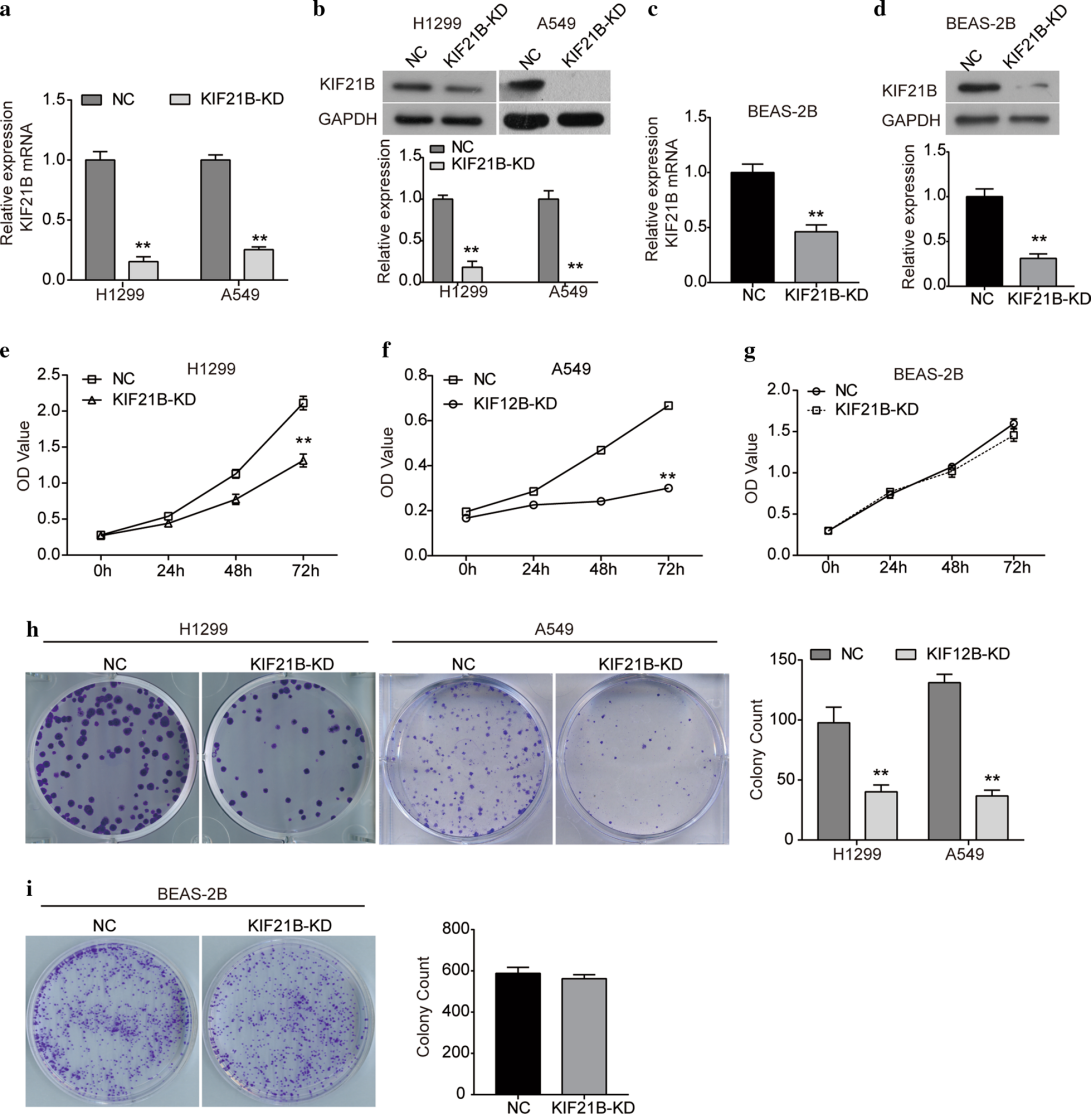
Knockdown of KIF21B inhibits the proliferation and colony formation abilities of NSCLC cells. H1299 and A549 cells were infected with shRNA-control lentivirus or sh-KIF21B lentivirus, as well as BEAS-2B cells. **a** qRT-PCR analysis of the expression of KIF21B mRNA in H1299 and A549 cells. **b** The expression of KIF21B protein in H1299 and A549 cells infected with sh-KIF21B lentivirus was detected by western blot. **c** qRT-PCR analysis of the expression of KIF21B mRNA in BEAS-2B cells. **d** The expression of KIF21B protein in BEAS-2B cells was detected by western blot. **e**–**g** CCK8 assay was performed to examine the viability of H1299 **e**, A549 **f** and BEAS-2B **g** cells after indicated transfection. **h** Colony formation abilities of H1299 and A549 cells were assessed by colony formation assay. **i** Colony formation ability of BEAS-2B cells was assessed by colony formation assay. NC, cells were infected with shRNA-control lentivirus; KIF21B-KD, cells were infected with sh-KIF21B lentivirus (KIF21B-knockdown). Data are presented as the mean ± standard deviation. Results were obtained in three replicates. **P < 0.01

Given the up-regulation of KIF21B expression in NSCLC tissues, in order to further investigate its biological function in NSCLC, we silenced the expression of KIF21B in NSCLC cell lines and normal BEAS-2B cells by infecting with shRNA-KIF21B-lentiviral. As indicated in Fig. 3a, b, the expression of KIF21B was obviously down-regulated in H1299 and A549 cells at both mRNA and protein levels. The expression of KIF21B was also reduced by sh-KIF21B in BEAS-2B cells (Fig. 3c, d). CCK8 assay showed that KIF21B knockdown reduced the proliferation abilities of both H1299 and A549 cells (Fig. 3e, f). However, silencing of KIF21B did not affect the proliferation of BEAS-2B cells (Fig. 3g). Further, we performed colony formation assay to assess the colony-forming abilities of H1299 and A549 cells after KIF21B was silenced. As shown in Fig. 3h, i, the results further confirmed that silencing of KIF21B inhibited the colony-forming abilities of H1299 and A549 cells, but not BEAS-2B cells.

### Knockdown of KIF21B promotes apoptosis and inhibits activation of the Akt signaling pathway in NSCLC cells

**Fig. 4:**
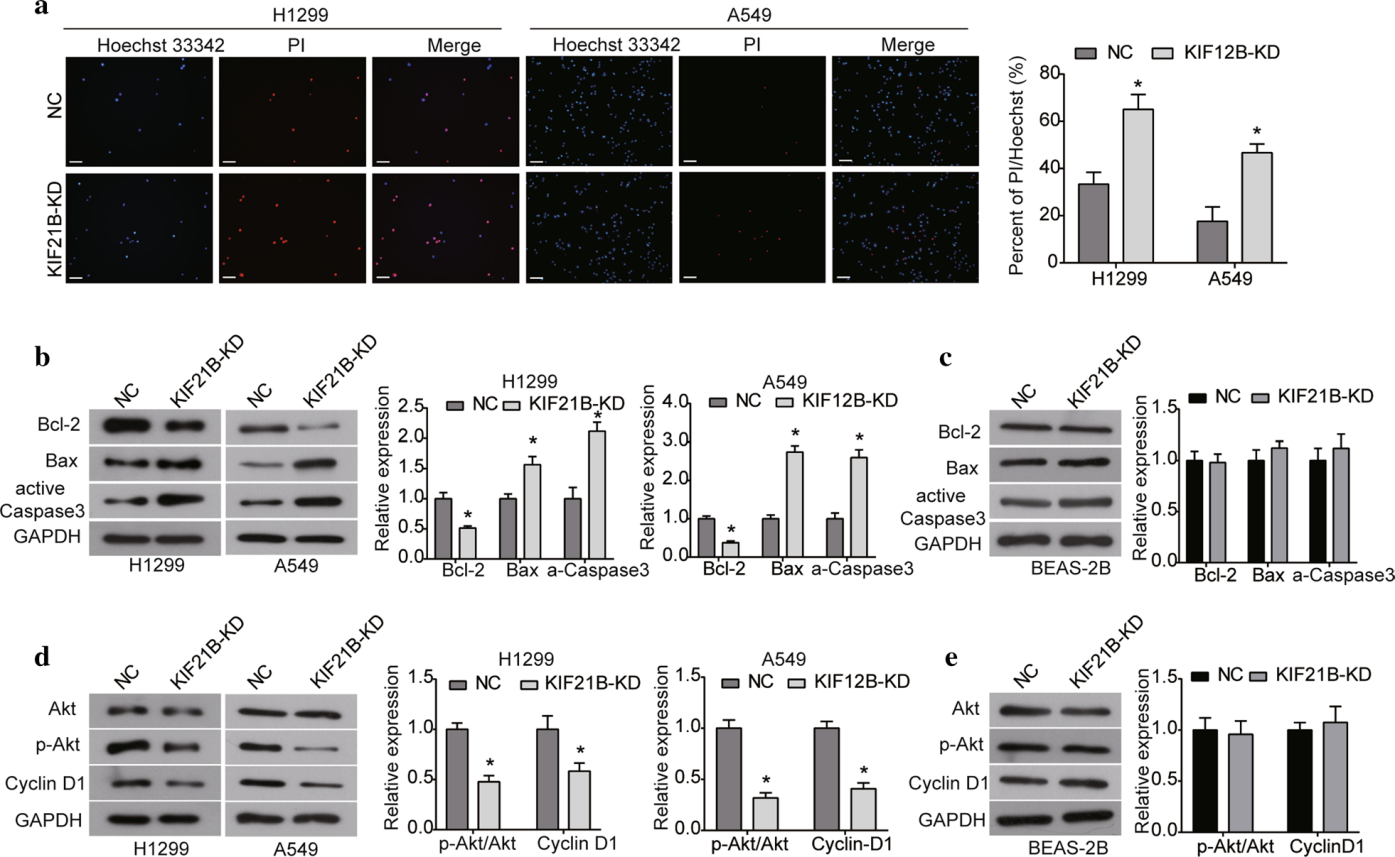
Knockdown of KIF21B promotes apoptosis in NSCLC cells. **a** After being infected with sh-KIF21B lentivirus, Hoechst 33342/PI assay was used to assess cell apoptosis in H1299 and A549 cells. The scale bar was 100 μm. **b** The expression of apoptosis-related proteins (Bcl-2, Bax and active Caspase 3) was detected by western blot in H1299 and A549 cells after indicated transfection. **c** The expression of apoptosis-related proteins was detected by western blot in BEAS-2B cells. **d** The expression of key components of the Akt signaling pathway (Akt, p-Akt and Cyclin D1) was examined using western blot in H1299 and A549 cells. **e** The expression of key components of the Akt signaling pathway was examined using western blot in BEAS-2B cells. Data are presented as the mean ± standard deviation. Results were obtained in three replicates. *P < 0.05

After being infected with sh-KIF21B for 72 h, cell apoptosis was determined using the Hoechst/PI assay. A significant increase in the percentage of apoptotic cells was observed in KIF21B-silenced cells compared with the NC group (Fig. 4a). Moreover, western blot assay revealed that the expression of Bcl-2 was significantly decreased in both H1299 and A549 cells after KIF21B was silenced, while the expression levels of Bax and active Caspase3 were increased (Fig. 4b). But we observed no significant effect of KIF21B knockdown on the expression levels Bcl-2, Bax and active Caspase 3 in BEAS-2B cells (Fig. 4c). Based on these data, silencing of KIF21B may promote apoptosis in NSCLC cells by modulating the Bcl-2/Bax expression and Caspase 3 activation.

Given the critical role of the Akt signaling pathway involved in the growth, metastasis and apoptosis of cancer cells, the effect of KIF21B on this signaling pathway was evaluated. Western blot demonstrated that KIF21B knockdown did not affect the level of total Akt in H1299 and A549 cells, but significantly decreased the phosphorylation form of Akt (p-Akt) (Fig. 4d). Consistently, the expression of downstream protein Cyclin D1 was inhibited by KIF21B knockdown in NSCLC cells (Fig. 4d). However, there was no significant change in the expression of Akt, p-Akt and Cyclin D1 in BEAS-2B cells after KIF21B was silenced (Fig. 4e).

### KIF21B inhibition suppresses the migration and invasion abilities of NSCLC cells

**Fig. 5:**
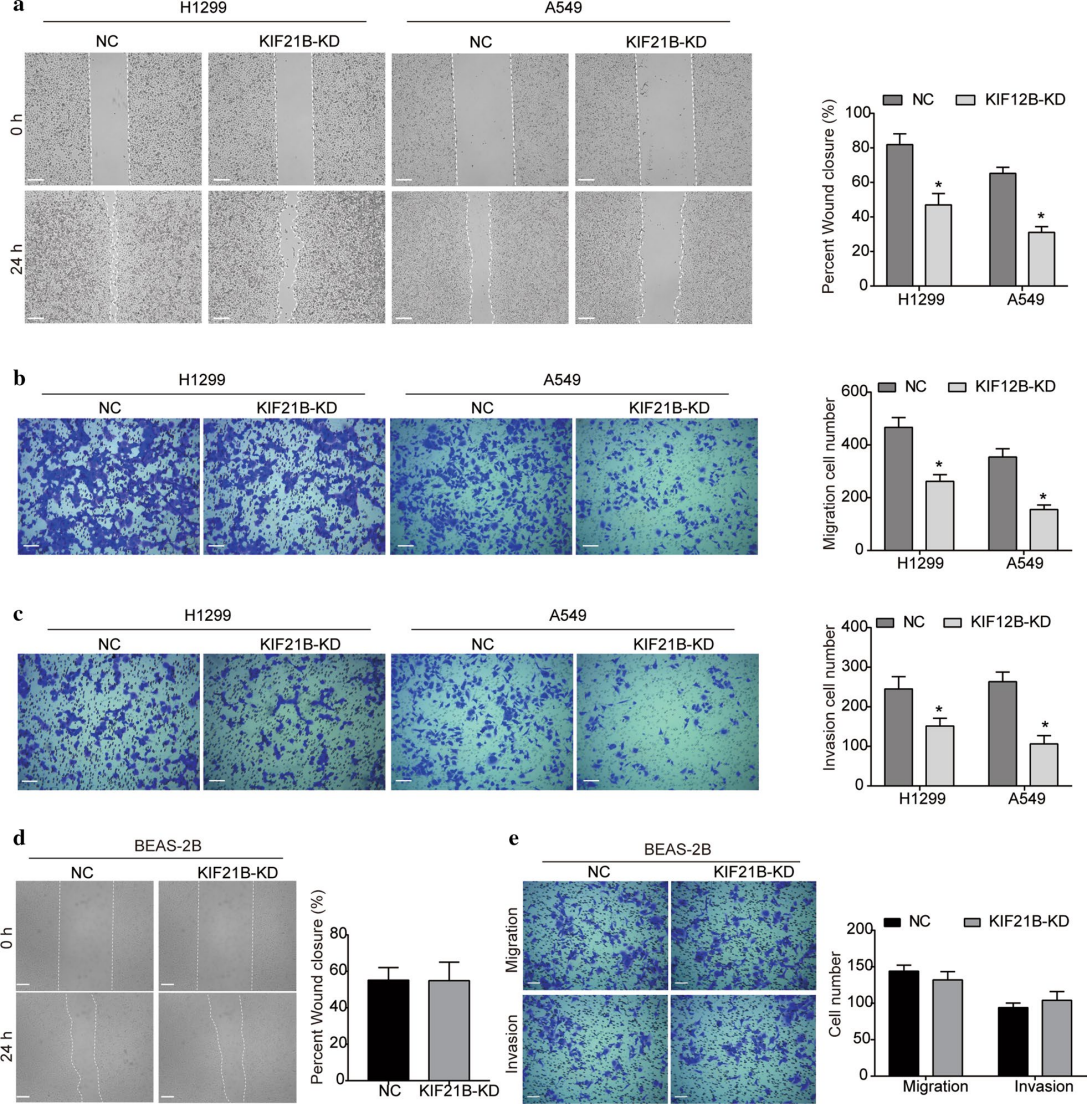
Silencing of KIF21B suppresses the migration and invasion abilities of NSCLC cells. **a** After being transfected with shRNAs, the effect of KIF21B knockdown on the migration of H1299 and A549 cells was assessed by wound-healing assay. **b, c** Transwell assay was performed for assessment of NSCLC cell migration **b** and invasion (**c**). **d** The effect of KIF21B knockdown on the migration of BEAS-2B cells was assessed by wound-healing assay. **e** Transwell assay was performed for assessment of the migration and invasion of BEAS-2B cells. Data are presented as the mean ± standard deviation. Results were obtained in three replicates. The scale bar was 100 μm. *P < 0.05

As indicated by wound-healing assay, the migration abilities of H1299 cells were significantly impeded after KIF21B was silenced (Fig. 5a). Similarly, the migration surface of A549 cells transfected with sh-KIF21B was also significantly decreased compared with the NC group (Fig. 5a). Additionally, results of transwell assay manifested that the number of migrated cells was significantly decreased by silencing KIF21B (Fig. 5b), which was further supported that KIF21B inhibition suppressed NSCLC cell migration. Moreover, in comparison with the NC group, H1299 and A549 cells infected with sh-KIF21B displayed a significant depression in cell invasion (Fig. 5c). Interestingly, silencing of KIF21B did not affect the migration and invasion capacity of BEAS-2B cells (Fig. 5d, e).

### Down-regulation of KIF21B inhibits xenograft growth in nude mice

**Fig. 6:**
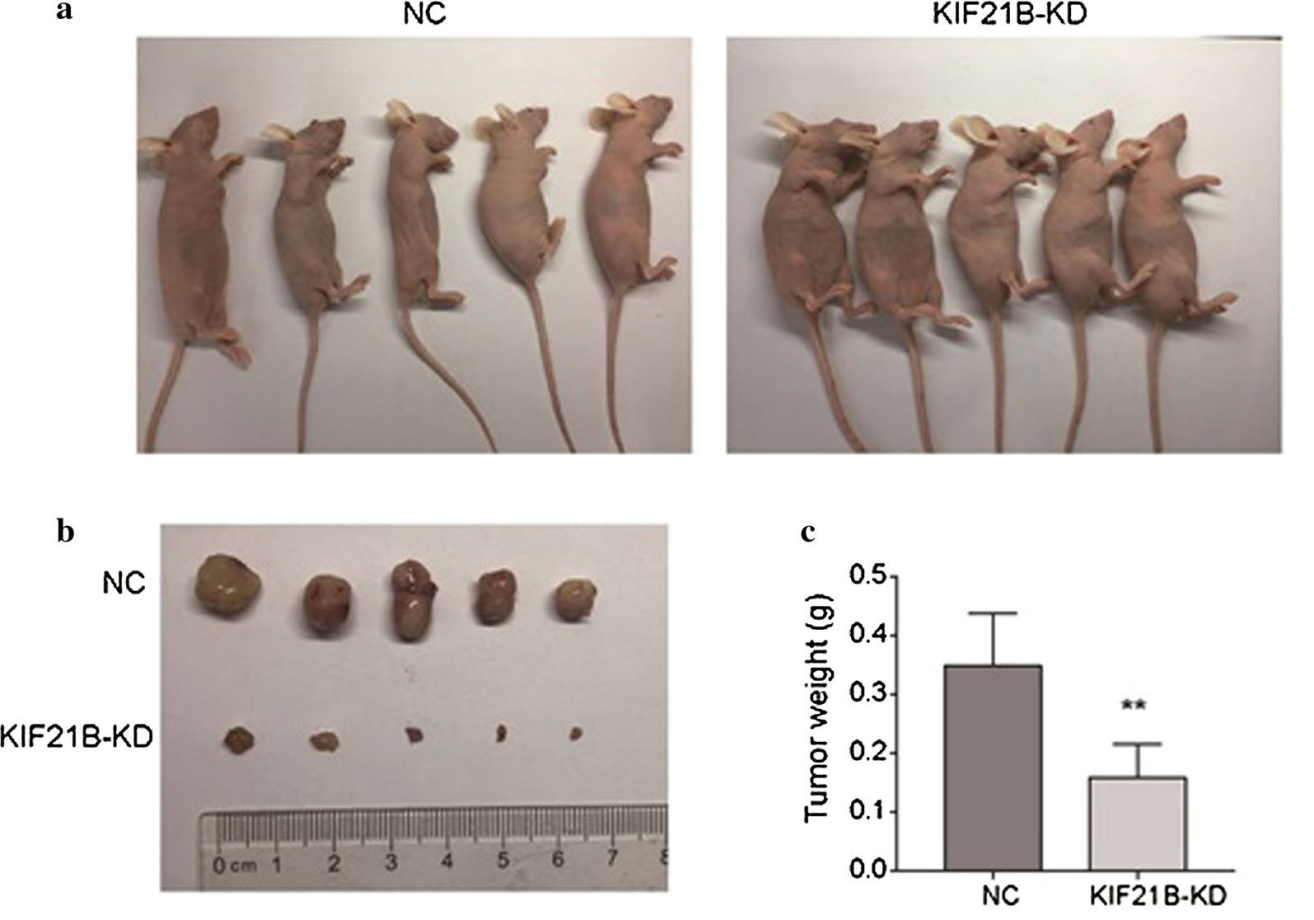
Knockdown of KIF21B inhibits tumor growth in nude mice. **a** The images of nude mice injected with H1299 cells transfected with shRNA-control lentivirus or sh-KIF21B lentivirus. **b**, **c** Representative xenograft tumors were shown **b** and the tumor weight was measured (**c**). **P < 0.01

Since the effects of KIF21B on NSCLC cells had been revealed by in vitro experiments, we further investigate its effect on tumor growth in vivo. The sh-NC and sh-KIF21B H1299 cells were subcutaneously (s.c.) xenografted into the BALB/c nude mice. The tumors were excised at the end of the experiment (Fig. 6a, b). Tumor weights were determined and plotted in Fig. 6c. Results showed that the tumor size and weight of KIF21B-silenced group were significantly lower than those of control group (Fig. 6a–c).

## Discussion

It is a generally acknowledged that lung cancer remains the main cause of tumor-related deaths globally, with about 1.4 million new cases diagnosed and approximately 1.6 million deaths per year worldwide [17, 18]. NSCLC is reported to account for nearly 85% of lung cancer. Tumor metastasis is observed in 79% of patients with NSCLC, and the 5-year survival rate of advanced patients is no more than 5% [19, 20]. Therefore, it is urgent to search for novel and reliable targets for NSCLC therapy.

In view of the critical roles of KIFs in mitotic cell division, accumulating evidence has demonstrated that KIFs are involved in tumorigenesis [21, 22]. For instance, previous reports revealed that up-regulation of KIF14 could promote cancer progression and metastasis, and could predict poor prognosis in gastric cancer and colorectal cancer [23, 24]. Gu et al. suggested that silencing of KIF26B suppressed breast cancer cell growth and invasion [25]. KIFC1 is also reported to be participated in the progression of NSCLC through regulation of cell proliferation and cell cycle [7]. Therefore, further understanding of the relationship between KIFs and tumor progression can provide a new perspective on the mechanism of tumorigenesis and serve effective targets for cancer therapy. Herein, our data highlight the oncogenic role of KIF21B in NSCLC.

Recently, Zhao et al. revealed that KIF21B was up-regulated in hepatocellular carcinoma tissues and cells, which was an independent risk factor for overall survival and disease-free survival in patients with hepatocellular carcinoma after hepatectomy [13]. In the present study, we determined that KIF21B was up-regulated in NSCLC tissues compared to adjacent normal tissues, which was consistent with the expression pattern of KIF21B in hepatocellular carcinoma. Moreover, high KIF21B expression was correlated with lymph node metastasis and tumor stage, predicting a poor prognosis of patients with NSCLC. Hence, based on these findings, KIF21B may function as a potential prognostic biomarker in NSCLC might be participated in the development of NSCLC.

It’s well known that uncontrolled proliferation is a prominent feature of malignant tumors. Gu et al. showed that depletion of KIF26B triggers cell cycle could arrest through regulating CyclinD1 to modulate cell proliferation in mammary cancer [25]. Liu et al. showed that knockdown of KIFC1 could reduce cell proliferation and trigger G2/M phase arrest in A549 and SPC-A1 cells [7]. It has been recently reported that silencing of KIF21B could inhibit the proliferation of hepatocellular carcinoma BEL-7404 cells [13]. In this study, the loss-of-function experiments manifested that silencing of KIF21B suppressed the proliferation of H1299 and A549 cells, and inhibited NSCLC tumor growth in vivo, which was consistent with the proliferation promoting effect of KIF21B in hepatocellular carcinoma cells. Moreover, we observed a significant increase in apoptosis in KIF21B-silenced cells. The Bcl-2 family serves as the key regulators of intrinsic apoptotic pathway which is the main mechanism of apoptosis, and the Bcl-2/Bax is a key regulator in determining cell fate [26]. Casapse 3 is another crucial executioner in apoptosis process [27]. We found that the expression of anti-apoptotic protein Bcl-2 was significantly down-regulated after KIF21B knockdown, while the expression of pro-apoptotic proteins Bax and active Casapse 3 was up-regulated. Therefore, these data suggested that down-regulation of KIF21B might promote apoptosis in NSCLC cells by regulating Bcl-2/Bax axis and Caspase 3 activation. Importantly, silencing of KIF21B did not affect the proliferation of BEAS-2B cells, as well as the apoptosis-related signaling. Taken together, these findings indicated an oncogenic role of KIF21B in NSCLC cell proliferation and apoptosis, and regulation of apoptosis by KIF21B might be involved in its regulation of NSCLC cell growth.

It has been widely held that the Akt signaling pathway, frequently over-activated in cancers, plays a central role in regulation of cellular processes and tumor progression such as proliferation and apoptosis [28]. Targeting Akt has been considered as an important approach for cancer prevention and therapy [29]. Therefore, to preliminarily explore the mechanism by which KIF21B affected NSCLC cell proliferation and apoptosis, we examined the effect of KIF21B knockdown on the Akt signaling pathway. Our data demonstrated that knockdown of KIF21B reduced activation of the Akt signaling pathway by decreasing the level of phosphorylation form of Akt in NSCLC cells, but did not affect the Akt signaling pathway in BEAS-2B cells. Additionally, the expression of Cyclin D1, a downstream protein of the Akt signaling pathway, was down-regulated in KIF21B-silenced cells. Cyclin D1 is revealed to function as a pivotal regulator of cell cycle, promoting the transition of cells from G1 to S phase [28]. Collectively, these results suggest that the Akt signaling pathway may be involved in the oncogenic role of KIF21B in NSCLC. Further, it is important to note that one of the leading causes of poor prognosis of cancer patients is tumor metastasis. Our data demonstrated that silencing of KIF21B decreased the migration and invasion of H1299 and A549 cells, but not BEAS-2B cells, suggesting the potential role of KIF21B in modulation of tumor metastasis. It has been confirmed that KIF26B regulates cell invasion in breast cancer through driving epithelial-mesenchymal transition (EMT) [23]. The effect of KIF21B on the EMT process in NSCLC will be further studied in our future research.

## Conclusion

In summary, for the first time, the current study demonstrated that KIF21B was up-regulated in NSCLC tissues, and its high expression was associated with poor clinicopathologic features and predicts a poor prognosis in NSCLC. Moreover, our data confirm that KIF21B exerts an oncogenic role in NSCLC progression, silencing of KIF21B can suppress the growth and invasion of NSCLC cells, as well as promoting apoptosis. Therefore, KIF21B may serve as a potential prognostic biomarker and therapeutic target for NSCLC.

## Data Availability

The data supporting the conclusions of this paper are included within the manuscript.
